# Supplementary material for: Learning curve and surgical outcome of robotic assisted colorectal surgery with ERAS program
Source: Sci Rep. 2022 Nov 29;12:20566. doi: 10.1038/s41598-022-24665-w (PMC9709162; doi:10.1038/s41598-022-24665-w)
Supplement: Supplementary file 2 — Supplementary Information 2. [file 41598_2022_24665_MOESM2_ESM.zip › RAL Raw data-Ñ[▒K/052/OP N_ñw╝╨░O▒Kñσ.pdf]

W102 / W102

臺中榮民總醫院
手術室護理記錄

[redacted]

基本資料

- 室別： 開刀房-17室，手術科別： CRS，手術類別： 常規，日期： 2021/01/16
- 進入時間： 0755 ； 麻醉開始時間： 0840 ； Time Out時間： 1025 ； 劃刀時間： 1026 ； 手術結束時間： 1620 送出時間： 1635 ；
- 麻醉方式： GE

  診斷： Malignant tumor of rectum SP CCRT

  手術名稱： Robot Assisted Low Anterior Resection

  手術局部用藥： NIL

  術前預防性抗生素：
  - 抗生素名稱一： Veterin 1GM IVD/由麻醉護理人員給予
  - 時間： 1015
  - 上次簽章: 許臺真

  ，追加預防性抗生素：
  - 抗生素名稱一： Veterin 1GM IVD/由麻醉護理人員給予
  - 時間： 1415
  - 上次簽章: 陳慧瑜
- 手術醫師： 蔣鋒帆，楊曙榮，呂曉瑜
- 麻醉醫師： VS 洪至仁
-
  - 刷手護士A ： 紀玉娟，時間： 0755，刷手護士B ： 陳慧瑜，時間： 0755，接替刷手一： 陳慧瑜，時間： 1200，接替刷手二： 紀玉娟，時間： 1230
  - 流動護士A ： 許臺真，時間： 0755，接替流動一： 陳慧瑜，時間： 1100，接替流動二： 陳怡君，時間： 1130
- 更多接替及流動護士： 接替刷手三： 彭芳渝，時間： 1500，接替流動三： 陳慧瑜，時間： 1230，接替流動四： 王昱瑀，時間： 1500

皮膚狀況

A-V Shunt： 無

術前： 不完整

部位(術前)：

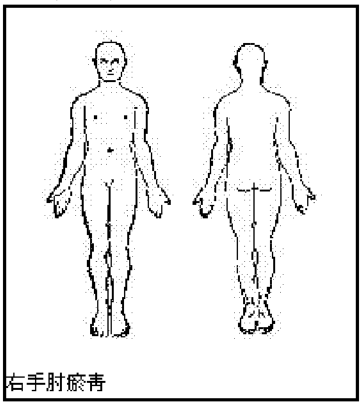

記錄者： 上次簽章: 許臺真

術後： 不完整

部位(術後)：

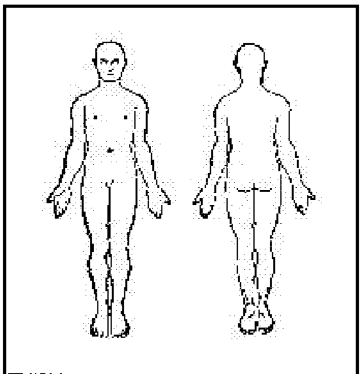

同術前

記錄者： 王昱瑀

手術準備

手術臥式 單一手術臥式

-
  - 手術臥式： 截石臥式
  - 使用附件： 溫毯 38 ℃，頭圈，腳架，其它： later supportX2,silicon rodX2
  - 皮膚準備： 10%Aq Betadine，Hibiscrub，2% Chlorhexidine
  - 電燒： 使用電燒: 電極片位置： 右大腿，雙極電燒，記錄者： 上次簽章: 許臺真

止血帶T： 無

植入物

- 植入物名稱/號碼/數量/部位： Davinci Xi EndoWrist Stapler:45mm x3
  Covidien GIA:80-3.5mm x1
  EEA:29mm/colon

, 記錄者： 上次簽章: 陳慧瑜

標本送檢

- 細菌培養 無
- 細胞學 無
- 冰凍切片 無
- 病理標本
  組織名稱一：rectum
  ,
  組織名稱二：IMA root
  ,
  組織名稱三：distal cutend
  ，更多組織名稱
  - 組織名稱四：peritonium
- 截肢 無

標本交付 標本： 4 袋，標本： 0 盒，標本： 0 瓶，細胞學： 0 管，Culture Tube： 0 管，被交付醫師： CR 楊曙榮，

- 記錄者： 王昱瑀
- 時間： 1630

傷口術後數料

傷口數料： 傷口數料： 無

- 請於圖上標示部位：

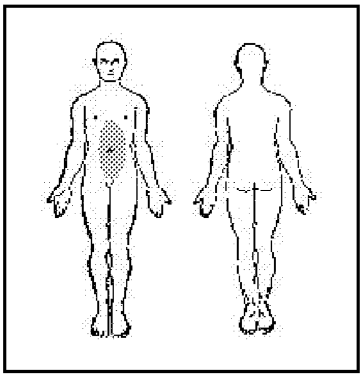

- 傷口敷料： guaze

導管

導管	天數	導管資訊	插入日期	插入時間	更換日期	拔除日期	拔除時間	拔除原因	預計拔除/更換	預計拔除/更換日期
Epidural PCA(1)	1	部位:硬脊膜;	2021/01/16	0845						
Foley(1)	1	號碼:16 Fr.; 部位:膀胱;	2021/01/16	0935						
A-line (1)	1	號碼:20 G; 部位:左手;	2021/01/16	0910						
Double Lumen CVC(1)	1	號碼:8 Fr; 深度:15cm; 部位:subclavian (右); 象限:RU;	2021/01/16	0900						

用物計數

- 手術中出血量： 100 c.c.
- 手術中尿量： 320 c.c.
-
  - 其它： 無

用物計數： 多切口同時縫合

- 1.器械： 正確，2.縫針： 正確，3.刀片： 正確，4.紗布： 正確，5.腹部墊： 未使用，6.紗球： 未使用，7.PEANUTS ： 未使用，9.絲帶： 正確，10.針頭： 未使用，11.Vessel loop： 未使用，12.Boots： 未使用，13.Jelco： 未使用，14.棉條： 未使用，
  - 第一次：
    - 刷手護士 ： 彭芳渝
    - 流動護士： 王昱瑂
    - 時間： 1530

  ,
  - 第二次：
    - 刷手護士 ： 彭芳渝
    - 流動護士： 王昱瑂
    - 時間： 1540

  ,
  - 第三次：
    - 刷手護士 ： 彭芳渝
    - 流動護士： 王昱瑂
    - 時間： 1550

備註

備註： Davinci scope 30度 NO.1

- 記錄者： 上次簽章: 許臺真
- 時間： 0755

填寫護理人員 王昱瑂

正式摘要

W102 / W102

doctorRank:B
